# Bacterial contamination of Ugandan paper currency notes possessed by food vendors around Mulago Hospital complex, Uganda

**DOI:** 10.11604/pamj.2018.31.143.16738

**Published:** 2018-10-25

**Authors:** Muhumuza Allan, Catherine Atuhaire, Musisi Nathan, Francis Ejobi, Samuel Nambile Cumber

**Affiliations:** 1 School of Biosecurity, Biotechnical and Laboratory Sciences, College of Veterinary Medicine, Animal Production and Biosecurity, Makerere University, Uganda; 2 Faculty of Medicine, Department of Nursing, Mbarara University of Science and Technology, Uganda; 3 School of Biosecurity, Biotechnical and Laboratory Sciences, Department of Biotechnical and Diagnostic Services, Makerere University, Uganda; 4 Faculty of Health Sciences, University of the Free State, Bloemfontein, South Africa; 5 Section for Epidemiology and Social Medicine, Department of Public Health, Institute of Medicine, The Sahlgrenska Academy at University of Gothenburg, Gothenburg, Sweden

**Keywords:** Bacterial contamination, pathogens, food vendors, Escherichia coli, Staphylococcus aureus, paper notes

## Abstract

**Introduction:**

Paper currency notes, exchangeable fomite, that is continuously contaminated because of the poor handling and storage practices. Objective: the general objective of the study was to determine the bacterial contamination of paper currency notes possessed by food vendors around Mulago National Referral Hospital Complex.

**Methods:**

A total of sixty paper notes of six denominations (1000, 2000, 5000, 10000, 20000, 50000) were collected from different food vendors. Each note was preserved in a sterile falcon tube and transported to the microbiology lab for bacteriological examination. Data from questionnaires was analyzed using SPSS version 23 (IBM SPSS Statistics).

**Results:**

All sampled paper notes had bacterial contamination. The bacterial counts ranged from 4×10^2^cfu/ml to 6.8×10^9^ cfu/ml, with the Shs.1000 notes having the highest average total bacterial load of 2.17×10^9^ cfu/ml and highest average total coli form counts of 21.5×10^2^ cfu/ml. The fifty thousand shillings note had no coliform detected. Of the analysed 60 samples, 27(45%) samples contained *Staphylococcus aureus*. None of the sampled paper notes had *Escherichia coli*.

**Conclusion:**

The study revealed that most of Ugandan paper notes are contaminated with bacteria including potential pathogens that cause disease in healthy individuals and opportunistic pathogens that may cause disease in hospitalized and immunocompromised patients. This study showed that the most contaminated note denominations were those of low denomination (Shs.1000 and Shs.2000) which had the highest bacterial count. The study revealed the paper currency notes were stored in different places where the commonest was the drawer and kept with different items, the commonest being pens. Hence, great care must be taken while handling money during the preparation and handling of food to avoid cross contamination.

## Introduction

Paper currency is the most needed material by each and everybody in a civilized society to attain a socio-economical class of getting basic needs useful to human [1]. Paper currency note is widely exchanged for goods and services in the entire global economic environment and has promoted trade in communities since its first introduction in China approximately 1,000AD [2]. Current currency paper notes are made from a special blend of linen, cotton and gelatin from animals for the surface coating of currency paper note with few segments of fiber and the mixture of all the materials used make them strong. However recently many nations are adopting the plastic polymer made currency transferring from paper made [3]. Studies have shown that polymer made currency notes often have lower bacterial count compared with the cotton-based 'paper' currency notes thus this might be due to various physicochemical parameters of the polymers [4, 5]. Paper currency notes have been proved by research that they act as an ideal breeding ground of microorganisms for several reasons such as the big surface area for organisms and organic debris to collect [6]. However much it is believed that the paper note currency is impregnated with a particular disinfectant to inhibit microorganisms but pathogenic microorganisms have been isolated from those paper notes [7]. Various research on money in different nations showed that bacterial contamination in Bangladesh revealed coliform contamination of 80% of thirty old two taka notes [8] and from 94% of one dollar bills pathogenic microorganisms were isolated [9]. Ninety six of one hundred currencies were found contaminated with bacteria such as *Escherichia coli, Staphylococcus aureus, Pseudomonas Species, Bacillus species and Salmonella typhi* [10]. The Potential public health threat that currency notes might act as fomites for cross contamination of potential microorganisms was suggested in the 1970s [11]. The use of paper currency with lower denomination notes receiving the most handling because they were exchanged frequently [12]. Studies show that the denomination of the currency notes and the age factor are directly correlated with the contamination observed where by older currency notes had the most contamination compared to newer ones [13].

Microbial contamination of currency notes could be from several sources, it could be from the production process, storage, counting machine, atmosphere, usage and handling [14]. The source of contamination could be as a result of poor money handling practices like spraying during the several events and ceremonies where such notes may be stepped on by dirty shoe soles when they fall on the ground [15]. Other practices like wetting the fingers with saliva from the mouth during counting paper currency notes could lead a possible transfer of potential pathogenic bacteria through a cross contamination [16]. Continuously, people squeeze paper currencies and push them in their unclean pockets, dirty stockings and some of the ladies put currency notes in the space between their two breasts. During food preparations like animal slaughtering, currency notes are handled with hands contaminated with blood and animal wastes when selling that meat [17, 18]. It is believed that simultaneous handling of food and paper currency notes adds to the chances of food related public health conditions [11]. According to the available data for the last two decades shows that pathogens on currency notes could represent a potential cause of food borne illness [19]. Unfortunately, many 'ready to eat' food selling points base on paper currency for exchange with high incidences of content between the currencies and food putting the consumer food safety at risk [20]. Studies done show that there is spread of food borne infectious agents through handling of contaminated paper currency notes and taking 'ready to eat' food [20]. Considering a hospital setting where food vendors selling food to health workers, patients as well as their caretakers in exchange of currency notes, increases the risks of cross contamination of potential pathogenic microorganisms like *Pseudomonas* species, *Staphylococcus, Micrococcus species, Escherichia coli* etc [10]. It is from that very incident that leads to the cause of food borne illnesses which have increased morbidity and mortality rates especially in populations of immune compromised people and children [19]. However, the bacterial contamination of paper currency notes possessed by food vendors who handle food for the public has not been well documented in Uganda and this becomes the gist of this research study.

**Broad objectives:** The study assessed the bacterial contamination of Ugandan paper currency notes possessed by food vendors in Mulago National Referral Hospital Complex.

**Specific objectives:** Determination of the total aerobic plate load of the paper currency notes; isolation and identification of *Staphylococcus aureus* on the paper currency notes; determination of the total coliform count on the paper currency notes; determination of the *Escherichia coli* count on the paper currency notes.

## Methods

**Study setting:** The study was conducted in Mulago National Referral Hospital Complex which is located on Mulago Hill in the northern part of the city of Kampala (capital city of Uganda) immediately west of Makerere University College of Health Sciences. It is approximately 5 kilometers by road, northeast of Kampala's central business. The coordinates of the hospital are Latitude: 0.337780 and Longitude: 32.575550. The reason for choosing this place was because it received almost new different people in masses and this increased chances of contaminating paper currency notes in circulation. This was a cross-sectional study which was carried out from February to April, 2018.

**Study units:** Paper currency notes of all denominations were collected from food vendors and transported aseptically from the study area to the laboratory for analysis.

**Sample size:** sample size determination for contamination of paper currency notes was estimated at 50% since the previous contamination in this area was not known, therefore by applying the formula according to Kish and Leslie (1998) the sample size (n) was obtained as below

$$\text{n}=\frac{Z\alpha^{2}\times \text{p}\left(1-\text{p}\right)}{d^{2}}$$

In the formula, n = sample size Z (Confidence interval) = 1.645 p (estimated prevalence) = 50% (0.5) d (allowable error) = 10% (0.1)

$$\text{n}=\frac{1.96^{2}\times 0.5\left(1-0.5\right)}{{\left(0.1\right)}^{2}}$$

n=67.65≈68 samples.

**Sampling technique:** According to the pilot study that was carried out around the study area, there are 40 kiosk canteens and food restaurants. Fifteen kiosk canteens and food restaurants were picked at random. All denominations of paper currency note (1,000, 2,000, 5,000, 10,000, 20,000, 50,000) were considered during collection from each of the kiosk canteen and food restaurants that was considered. A total of 60 paper currency notes were sampled. The samples were collected aseptically by engaging food vendors with questionnaires to drop the paper currency into sterile falcon tubes. The sterile falcon tubes were promptly sealed and individuals were given a replacement note equivalent to the denomination they had deposited in the sampling falcon tube(s). The falcon tubes were immediately transported aseptically to the laboratory for microbial analysis.

**Laboratory methods:** Microbial analysis of paper currency notes was carried out on arrival in the Laboratory, sample IDs were recorded in the Laboratory book for easy tracking prior to processing.

**Preparation of media:** Media used was MacConkey, Plate Count Agar, Salt Nutrient Agar. Media preparation was done following the manufacture's instruction found on the media bottles. Appropriate amounts of powder media were weighed then placed in conical flask and the appropriate amount of distilled water were added to dissolve the powder. The media was sterilized using and autoclave at 121°C for 15 minutes. Media was allowed to cool before it was poured into Petri dishes arranged on a level surface.

**Determination of total aerobic plate count:** The sample of paper currency note was placed in 10ml of sterile peptone water, agitated using a vortex machine for 2 minutes to wash all contents of contamination from the paper note and the resulting solution was added with 90ml of sterile normal saline. 1ml of the resulting solution was serially diluted up to 10^-3^ magnitude. 0.1ml of sample homogenate from serial dilutions were introduced onto sterile plate count agar (Oxford, UK) plates. Dilutions in the two tubes were plated on the plate count agar by surface spreading using a sterile glass spreader. The plates were labeled using a marker and then incubated at 37°C for 24 hours to 48 hours to allow growth. After 24 hours the number of colonies were counted using a colony counter which were converted to colony forming units using the formula below;

$$\text{Colony}\;\text{Forming}\;\text{Units}\;=\;\frac{\text{number}\;\text{of}\;\text{colonies×dilution}\;\text{factor}}{\text{volume}\;\text{of}\;\text{culture}\;\text{plate}}$$.

**Determination of total coliform count:** The sample of paper currency note was placed in 10ml of sterile peptone water, agitated using a vortex machine for 2 minutes to wash all contents of contamination from the paper note and the resulting solution was added with 90ml of sterile normal saline. 1ml of the resulting solution was serially diluted up to 10^-1^ magnitude. The 1 in 10 ml dilution was cultured on MacConkey agar by surface spreading using a sterile glass spreader. The plates were then incubated at 37°C for 24 hours. The pink colonies ie coliforms after incubation were counted and expressed as colony forming units.

$$\text{Colony}\;\text{Forming}\;\text{Units}\;=\;\frac{\text{number}\;\text{of}\;\text{colonies×dilution}\;\text{factor}}{\text{volume}\;\text{of}\;\text{culture}\;\text{plate}}$$

**Determination of *Escherichia coli* count on paper currency:** The sample of paper currency note was placed in 10ml of sterile peptone water, agitated using a vortex machine for 2 minutes to wash all contents of contamination from the paper note and the resulting solution was added with 90ml of sterile normal saline. 1ml of the resulting solution will then be serially diluted up to 10^-1^ magnitude. The 1 in 10 ml dilution was cultured on MacConkey (Himedia, UK) agar by surface spreading using a sterile glass spreader. The plates were incubated at 44^0^C for 24-48 hours. The pink flat colonies with entire marginie coliforms after incubation were counted and expressed as colony forming units. Colony Forming Units = (number of colonies ×dilution factor)/(volume of culture plate) Confirmation of *E.coli* was done by carrying out a Gram staining technique followed by a biochemical test. For Gram staining technique, a slide was labeled on the frosted side using a pencil, a smear made from an emulsified colony, air dried and heat fixed. The fixed smear was covered with crystal violet stain for 30-60 seconds then washed off with clean water. The water was tipped off and the smear covered with lugol's iodine for 30-60 seconds then washed off with clean water. The smear was decolorized with acetone alcohol and immediately washed off with clean water. The smear was covered on with neutral red stain/dilute carbofuschin for 2 minutes and washed off with clean water. The back of the slide was wiped with a clean piece of cloth and placed on a rack for drying. The smear was examined microscopically under 40 × objective to check the staining and see the distribution and then with oil immersion objective to report the observed bacteria with a gram negative stain with a rod shape [21]. For the biochemical test, Indole test using Kovacs reagent was used. Whereby a discrete colony was obtained and emulsified in sterile peptone water containing tryptophan and incubated overnight for about 16-24 hours at 37°C. When the peptone water turned cloudy, then the test was performed as follows; 1ml of the culture was transferred to an empty glass tube, and then five drops of xylene were added following the adding of five drops of Erhlich's reagent. The mixture was agitated and left to stand for about 3 minutes and the positive result was shown by the presence of a red or red/violet colour in the surface alcohol layer of the broth [21].

**Isolation and identification of *Staphylococcus aureus*:** The sample of paper currency note was placed in 10ml of sterile peptone water, agitated using a vortex mixer for 2 minutes to wash all contents of contamination from the paper note. Using a sterile wire loop, it was soaked in the sample liquid and by use of streaking method on Salt Nutrient Agar (Oxoid, UK) the sample was cultured by incubating at 37°C for 24-48 hours. Yellow small colonies were identified. Confirmation of *Staphylococcus aureus* was done by carrying out a Gram staining technique followed by a biochemical test. For Gram staining technique, a slide was labeled on the frosted side using a pencil, a smear made from an emulsified colony, air dried and heat fixed. The fixed smear was covered with crystal violet stain for 30-60 seconds then washed off with clean water. The water was tipped off and the smear covered with lugol's iodine for 30-60 seconds the washed off with clean water. The smear was decolorized with acetone alcohol and immediately washed off with clean water. The smear was covered on with neutral red stain / dilute carbolfuchsin for 2 minutes and washed off with clean water. The back of the slide was wiped with a clean piece of cloth and placed on a rack for drying. The smear was examined microscopically under ×40 objective to check the staining and see the distribution and then with oil immersion objective to report the observed bacteria with a gram positive stain, round shaped in clusters [21]. For the biochemical test, coagulase test was done by picking a colony and homogenizing it in normal saline on a glass slide before a drop of rabbit plasma anticoagulated with Ethylenediaminetetraacetic acid (EDTA) were added. The slide was rocked gently for about 30seconds. The development of clumping within 10 seconds was recorded as positive reaction.

**Quality control and quality assurance:** Quality control is a reactive process with a set of activities used to verify quality of the output. Quality assurance is a proactive process of managing quality. A number of measures were put in place during sampling, microbial and statistical analysis to ensure production of quality results. Samples were placed in sterile polythene bags and culturing was done under maximum aseptic conditions for example the media, wire loop, Petridish all were sterilized before use. The workplaces were disinfected with 70% alcohol before and after work. All plates were checked for sterility before use by incubating at 37°C then overnight and checking for any bacterial growths. Samples were labeled to avoid errors thus increased accuracy. Laboratory standard operating procedures were followed to maintain accuracy and consistency. Work was done under maximum supervision.

**Data management and analysis:** Data was collected and entered into logbooks which were kept in a bookshelf. Computerized data was entered into excel software program keeping softcopy always. Data from questionnaires was analyzed using SPSS version 23 (IBM SPSS Statistics). Comparison of data was carried out between various denominations of currency and statistical data analysis involved descriptive analysis of bacterial isolates, colony forming units where by bar charts and frequency distribution tables were drawn.

**Ethical considerations:** Ethical clearance was obtained from the Ethics and Research Committee, Makerere University through the College of Veterinary medicine, Animal Resources and Bio-security, (Ethical Clearance Number 15/U/8035/EVE), Uganda. To gain access to information from respondents the letter was received from the Director of Makerere University so as to get access to the respondents. Participation was voluntary and informed written consent was provided by each respondent. The right to decline or to withdraw from the study at any stage without incurring any penalty was explained. All data in the questionaires were coded and kept locked up, accessible only to the researcher. Codes were used instead of subject names that ensured confidentiality of their initials. There were no risks or adverse effects encountered.

**Limitations of the study:** Some of the short comings during the study was fear and refusal of food vendors to give away their paper currency notes but this was overcome by presenting myself professional with a University ID for identification, a letter from the school Dean and use of a questionnaire to create a relationship with the respondent.

## Results

majority of samples had bacterial contamination. The microbial counts ranged from 4×10^2^ to 6.8×10^9^ cfu/ml. All the samples exhibited varying total aerobic plate counts, with the one thousand shilling notes having the highest average total aerobic plate counts of 2.17×10^9^ cfu/ml The coliform counts ranged between 1×10^2^ and 8×10^2^cfu/ml, with the one thousand shilling notes having the highest average total coliform counts of 21.5×10^2^ cfu/ml and the fifty thousand shilling notes having no count (Table 1).

**Table 1:** Mean bacterial load and average coliform counts of each denomination of paper currency notes

Paper Note (Ugshs)	Average TPC (cfu/ml)	Average TCC (cfu/ml)
1000	2.17×10^9^	21.5×10^2^
2000	7.14×10^8^	14.5×10^2^
5000	4.29×10^8^	3×10^2^
10000	9.51×10^8^	4.5×10^2^
20000	2.26×10^6^	5×10^2^
50000	6.81×10^8^	0

Key: TPC – Total Plate Counts and TCC – Total Coliform Counts

The paper currency notes were graded using condition, appearance and degree of dirtiness as new, moderate and old depending on the year of printing as shown (Table 2).

**Table 2:** Physical conditions of Ugandan paper currency notes collected from food vendors

Condition	Paper currency note	Number of notes per year of printing			Total
		2015 (OLD)	2016 (MODERATE)	2017 (NEW)	
Clean	1000	0	0	2	2
	2000	4	0	3	7
	5000	0	3	5	8
	10000	2	2	4	8
	20000	0	3	6	9
	50000	2	6	2	10
	TOTAL	8	14	22	44
	1000	5	2	1	8
	2000	0	0	3	3
	5000	2	0	0	2
	10000	1	0	1	2
	20000	0	1	0	1
	50000	0	0	0	0
	TOTAL	8	3	4	16
Grand Total					60

During sample collection, the vendors kept paper currency notes in different places with different items as shown in Table 3.

**Table 3:** Table shows different places where food vendors used to keep paper currency notes with different items

Places where paper currency notes were kept	Items kept with papercurrency notes						
	Coins	Haircombs	Keys	Pens	Phone	None	Total
Bucket	0	0	0	2	0	0	2
Drawer	6	1	3	6	0	3	23
Food Packets	1	0		0	0	0	1
Hand Bag	0	0	0	1	0	0	1
Handkerchief	0	0	0	0	0	1	1
Pocket	0	0	1	1	1	0	2
Total	7	1	4	10	1	4	30

**Figure 1:**
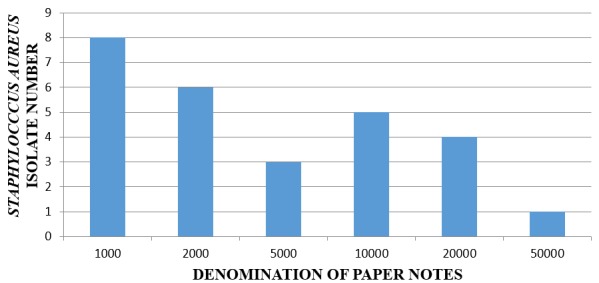
A bar chart showing the paper currency notes with number of Staphylococcus aureus isolated from each

Of the 60 analysed samples, 27(45%) samples contained *Staphylococcus aureus* and none of the sample had *Escherichia col*i (Figure 1).

## Discussion

In Uganda, poor currency handling culture is widespread, and there is an indiscriminate abuse of currency notes. Majority of the community people do not carry money in wallets and squeeze the notes in common occurrence. Women are especially among the unenlightened, often place underneath their brassieres, while men place it in their socks. These activities not only enhance currency contamination but may also increase the risk of infection from contaminated notes [14]. In similar study conducted by [22], a total of 176 samples of the examined 200 one Riyal notes had mixed bacterial growth. The present study documented the isolation and identification of *Staphylococcus aureus* bacteria from Ugandan paper currency notes and these bacteria could act as potential pathogens to human. Until recently worldwide studies concerning the bacterial contamination of paper notes have been few and rather limited, this was reflected on the scarcely available literature and this study consisted the first trial to assess the bacterial contamination of some Ugandan paper currency notes from various denominations in circulation in Mulago Hospital Complex. Currency notes in circulation last for years before being withdrawn, during this period they may habour various types of pathogenic, opportunistic and non-pathogenic bacteria [23]. Prolonged stay of paper currency notes in circulation increases the chances of the notes to be more contaminated [24]. These contaminants include pathogenic organisms which cause disease in healthy individuals as well as bacterial that cause diseases for hospitalized and immuno-compromised patients [19].

The method used in this study to isolate bacteria containing Paper currency notes was found suitable, reliable and could be conveniently used in future studies. This method was a modification of that of [9], who successfully isolated different types of bacteria from paper notes. All denominations investigated ( Shs. 1,000, 2,000, 5,000, 10,000, 20,000, 50,000) revealed bacterial growth, The one thousand shillings note had the highest total bacterial count followed by Shs. 10000, Shs.2000, Shs.50000, Shs. 5000, Shs.20000 respectively. The findings were similar to results of [23], who found that one dollar bill had the highest bacterial count since it was the lowest denomination and being the commonly handled note. The Sh.1000 and Shs.2000 notes were found to have the higher number of *Staphylococcus aureus* isolates (29.6%) and (22.2%) respectively. These high bacterial counts and high rate of isolation in smaller denominations may be due to the fact smaller denominations are more frequently used and exchanged, and accordingly liable to more contamination as suggested by [23]. In this study, there was no *E.coli* detected on paper currency notes but most frequently isolated bacterium was *Staphylococcus aureus* with isolation rate of 45% which is normal inhabitant of the human skin, it was also isolated by [9, 10, 23]. According to the study, paper currency notes were kept in different places by different vendors which included buckets, drawers, handkerchiefs, food packets, handbags etc and different items like keys, pens, coins, hair combs, phones shared storage space with paper notes. This goes a long way to reveal the poor sanitary conditions of the environment as well as poor hygiene practices that could have contributed to the Coliform counts detected. These items could get contaminated and spread the pathogens to the user through cross contamination [25].

## Conclusion

The study has revealed that most Ugandan paper note are contaminated with bacteria including potential pathogens that cause disease in healthy individuals and opportunistic pathogens that may cause disease in hospitalized and immuno-compromised patients. The most common isolated bacteria were *Staphylococcus aureus* which is a potential pathogenic microorganism. This study showed that the most contaminated note denominations were those of low denomination (Shs.1000 and Shs.2000) which had the highest bacterial count. The study further revealed the paper currency notes were stored in different places where the commonest was the drawer and kept with different items, the commonest being pens.

**Implication to practice:** Currency notes must be handled with caution; improvement of personal hygiene to reduce the extent of contaminating paper notes; hands should be washed carefully after handling currency notes; food vendors should be educated and have awareness to avoid possible cross contamination between currency notes and food by avoiding handling currency notes as they sell; incorporation of antimicrobial substances into paper films to reduce the level of bacterial contaminations

**Implication to policy:** Shortening the duration of circulation of paper notes; expand the usage of Credit Cards(C.C) as an alternate to currency notes; regular microbial testing of currency notes and establishment of method for large scale replacement of contaminated currency shall be employed; embrace the upcoming forms of digital currencies like Crypto currency such as Bit coin, One coin; introduction of plastic currency notes which can be washed easily as in Australia can serve as an alternate.

**Future research:** A more complex study using molecular techniques would be required to prove transmission of bacterial diseases from person to person via paper currency; further work is required to provide complete picture about the extent of the contamination of the other denomination in circulation like coins; more study on the antimicrobial resistance patterns of the Staphylococcus aureus species that were isolated

### What is known about this topic

- Paper currency notes have been proved to act as an ideal breeding ground of microorganisms;
- Microbial contamination of currency notes could be from several sources like production process, storage, counting machine, atmosphere, usage and handling;
- Simultaneous handling of food and paper currency notes adds to the chances of food related public health conditions.

### What this study adds

- This marks as the first study in Uganda on the bacterial contamination of Ugandan paper currency notes;
- It will give knowledge to the public on the potential public health threat of paper currency notes containing potential pathogenic microorganisms.

## Competing interests

The author declare no competing interests.
